# Radiative and Non-Radiative Decay Pathways in Carbon Nanodots toward Bioimaging and Photodynamic Therapy

**DOI:** 10.3390/nano12010070

**Published:** 2021-12-28

**Authors:** Yujin Kim, Yoonsang Park, Seulgi Han, Wonchan Park, Mungu Kim, Kyunghwan Kim, Jinmyoung Joo, Sei Kwang Hahn, Woosung Kwon

**Affiliations:** 1 Department of Chemical and Biological Engineering, Sookmyung Women’s University, Seoul 04310, Korea; eugene5980@sookmyung.ac.kr (Y.K.); ypark@keti.re.kr (Y.P.); 2 Institute of Advanced Materials and Systems, Sookmyung Women’s University, Seoul 04310, Korea; 3 Department of Materials Science and Engineering, Pohang University of Science and Technology (POSTECH), Pohang 37673, Korea; seulgihan@postech.ac.kr (S.H.); mlblucky@postech.ac.kr (W.P.); kimmungu@postech.ac.kr (M.K.); 4 Department of Chemistry, Ulsan National Institute of Science and Technology (UNIST), Ulsan 44919, Korea; starcato@unist.ac.kr; 5 Department of Biomedical Engineering, Ulsan National Institute of Science and Technology (UNIST), Ulsan 44919, Korea; jjoo@unist.ac.kr

**Keywords:** carbon dot, near-infrared, reactive oxygen species, bioimaging, photodynamic therapy

## Abstract

The origin and classification of energy states, as well as the electronic transitions and energy transfers associated with them, have been recognized as critical factors for understanding the optical properties of carbon nanodots (CNDs). Herein, we report the synthesis of CNDs in an optimized process that allows low-temperature carbonization using ethanolamine as the major precursor and citric acid as an additive. The results obtained herein suggest that the energy states in our CNDs can be classified into four different types based on their chemical origin: carbogenic core states, surface defective states, molecular emissive states, and non-radiative trap states. Each energy state is associated with the occurrence of different types of emissions in the visible to near-infrared (NIR) range and the generation of reactive oxygen species (ROS). The potential pathways of radiative/non-radiative transitions in CNDs have been systematically studied using visible-to-NIR emission spectroscopy and fluorescence decay measurements. Furthermore, the bright photoluminescence and ROS generation of these CNDs render them suitable for in vitro imaging and photodynamic therapy applications. We believe that these new insights into the energy states of CNDs will result in significant improvements in other applications, such as photocatalysis and optoelectronics.

## 1. Introduction

Over the past few decades, carbon nanodots (CNDs) have been recognized as luminescent materials with properties related to photostability, biocompatibility, and bioavailability. These properties enable CNDs to be used in the biomedical field for potential applications in bioimaging [1,2,3,4,5], biosensing [6,7,8,9], gene/drug delivery [10,11,12,13], and other nano-therapeutics [14,15]. Depending on the synthetic procedures and conditions, different core structures with various surface functional groups can be obtained for CNDs, endowing them with unique optical properties such as broadband and excitation-dependent photoluminescence (PL).

Radiative recombination or PL pathways in CNDs have been suggested in several studies: (i) carbogenic core states [16,17,18], (ii) surface defective states [19,20,21], and (iii) molecular emissive states [22,23,24]. The interactions between these PL pathways are strongly dependent on the chemical structure of CNDs; however, a comprehensive PL mechanism of these pathways remains unclear and controversial. One possible reason for this is that various preparation methods for CNDs lead to intrinsically different chemical structures, making any comparison between different CNDs almost impossible. Therefore, various experimental parameters that affect the optical properties of CNDs have been discovered, such as reaction temperature [25,26,27], heteroatom doping [28,29,30], and surface passivation [31,32,33]; however, further investigations are needed to evaluate the effects of these parameters.

Various types of CNDs and their chemical structures have been widely studied. Particularly, CNDs produced by the carbonization of organic acids and amines have been studied due to their ease of synthesis and high reaction yields. According to Song et al., molecular fluorophores with molecular emissive states are expected to form at low temperatures, whereas states with surface defects are predominantly formed at high temperatures [34]. Schneider et al. reported that the use of different amine precursors could change the PL intensity due to the formation of different molecular fluorophores [35]. The following areas of investigation emerge from these studies: (a) the reaction conditions required to form different types of chemical structures and energy states, (b) potential interactions occurring between the energy states, and (c) effect of these interactions on radiative and non-radiative transitions.

In this work, we report the synthesis of CNDs at relatively low temperatures (<100 °C) and investigate the generation of different energy states by controlling the addition of ethanolamine (EA) and citric acid (CA). Results obtained herein suggest that CNDs have four different types of energy states: carbogenic core states, surface defective states, molecular emissive states, and non-radiative trap states. Different types of emissions associated with each of these energy states and the potential pathways of radiative/non-radiative transitions have been thoroughly studied using visible-to-near infrared (NIR) PL spectroscopy and fluorescence decay measurements. Finally, we have assessed the potential of CNDs for in vitro imaging and photodynamic therapy (PDT) of adenocarcinomic human alveolar basal epithelial (A549) cells.

## 2. Experimental Section

### 2.1. Reagents

Citric acid and ethanolamine were purchased from Sigma-Aldrich (St. Louis, MO, USA). All chemicals were used as received without further purification.

### 2.2. Synthesis of CNDs

The synthetic procedure for the synthesis of CND1 is as follows: Ethanolamine (2 mL) was first vigorously stirred at 90 °C in air for 24 h. After the allotted time, the solution was allowed to cool to room temperature. The black sticky liquid was dissolved in water and dialyzed against water for at least 3 days using Spectra/Por Biotech Cellulose Ester dialysis tubes (Spectrum Chemical Mfg. Corp., Gardena, CA, USA) (100–500 Da). After the water was removed from the solution by lyophilization, the resulting powder was stored in a freezer for further use. The same procedure was followed for the synthesis of CND2, CND3, and CND4. The amounts of aqueous citric acid solution (1 g·mL^−1^) added to ethanolamine (2 mL) for CND2, CND3, and CND4, were 200 μL, 510 μL, and 2000 μL, respectively. The reaction yield of CNDs was ca. 100 mg.

### 2.3. Material Analysis

Transmission electron microscopy (TEM) was performed using a Jeol JEM-2200FS instrument (Jeol Ltd., Tokyo, Japan) equipped with a Cs corrector. High-performance liquid chromatography (HPLC) was performed using the following Shimazdu equipment (Shimadzu Corp., Kyoto, Japan): SIL-20A autosampler, LC-20AD pump, SPD-20A dual λ absorbance detector, and Shim-pack GIS-ODS 5 μm column. A 1:1 vol% mixture of water and methanol (flow rate: 0.8 mL·min^−1^) was used as the mobile phase. The injection volume was 25 μL. The CNDs were detected at wavelengths of 350 nm. Dynamic light scattering (DLS) and zeta potential measurements were performed using a Horiba SZ-100 particle-size analyzer (Horiba Ltd., Kyoto, Japan).

### 2.4. Chemical Analysis

X-ray photoelectron spectroscopy (XPS) was performed using an Escalab 250 spectrometer with an Al X-ray source (1486.6 eV) (VG Scientific Corp., Beverly, MA, USA). Fourier-transform infrared (FT-IR) spectroscopy was performed using a Nicolet iS50 FT-IR spectrometer (Thermo Scientific Corp., Madison, WI, USA). Raman spectroscopy was performed using a XperRam S spectrometer with 532 nm laser excitation (Alvatek Ltd., Tetbury, Glos, UK).

### 2.5. Optical Analysis

For spectroscopic measurements, 10 mm × 10 mm QS-grade quartz cuvettes (Hellma Analytics 111-QS) were used. UV-vis absorption spectra were recorded on a Scinco S-3100 spectrophotometer (Scinco Co. Ltd., Seoul, Korea). The PL spectra were recorded using a Jasco FP-8500 fluorometer (Jasco Inc., Tokyo, Japan). The absolute quantum yields were recorded on a Jasco FP-8500 fluorometer equipped with a 100 mm integrating sphere (ILF-835) and calculated using Jasco Spectra Manager II Version 2 software (Jasco Inc., Tokyo, Japan). NIR PL spectroscopy was performed using a HORIBA PTI QM-500 spectrometer (Horiba Ltd., Kyoto, Japan) with a conventional diode laser as the light source (660 nm, 1 W). Time-correlated single photon counting (TCSPC) measurements were performed using HORIBA Fluorolog-3 (Horiba Ltd., Kyoto, Japan); the emitted photon signal was spectrally dispersed by a monochromator and then collected using a fast photon multiplier tube detector.

### 2.6. ROS Measurement

Singlet oxygen sensor green (Thermo Fisher Scientific, Waltham, MA, USA) was used for the detection of singlet oxygen formation. The singlet oxygen sensor green was dissolved in 33 μL of methanol in order to form a solution of a concentration of 5 mM. 4 μL of the stock solution was added in 10 mL pH 7 buffer solution (Daejung Chemicals & Metals Co. Ltd., Siheung, Gyeonggi, Korea). The CNDs were dispersed in 2 mL of the solution at concentration of 0.1 mg·mL^−1^ in a quartz cuvette. After exposure to a UV lamp (254 nm, 8 W) for 2 h, the fluorescence was determined and excitation wavelength of 504 nm and emission wavelength of 515~600 nm.

### 2.7. Cell Viability Test

The cytotoxicity of the CNDs in FL83B and A549 cells were evaluated using the cell counting kit-8 (CCK-8) assay. The cells (1 × 105 cells/mL) were suspended in high-glucose Dulbecco’s Modified Eagle Medium (DMEM) and supplemented with 1 wt% antibiotic-antimycotic solution and 10 vol% FBS. Subsequently, the cells were seeded in each of the 96 wells of the cell culture plates (100 μL/plate) for 1 d. The CNDs were dispersed in a serum-free medium (SFM) at concentrations of 0.125, 0.25, 0.5, and 1 mg·mL^−1^, and were added to each well. The cell culture plates were incubated at 37 °C in a humidified 5% CO_2_ incubator for 1 d. Subsequently, the cells were washed with fresh phosphate-buffered saline (PBS) twice and treated with 10% CCK-8 assay solution. After an incubation period of 2 h, the optical density of each well was measured at 450 nm using a microplate reader (Molecular Devices, Sunnyvale, CA, USA). The experiment was repeated four times.

### 2.8. In Vitro Confocal Microscopy Imaging

Cell imaging was performed using confocal microscopy on FL83B and A549 cells. FL83B and A549 cells were prepared at a concentration of 1 × 10^5^ cells/mL in high-glucose DMEM complete medium and seeded (200 μL) in each chamber of the 8-chamber confocal slides for 24 h. The medium was replaced with 200 μL fresh SFM containing 1 mg·mL^−1^ of CNDs and incubated at 37 °C in a humidified 5% CO_2_ incubator for 1 d. Subsequently, the cells were washed with PBS twice and fixed with a 4% formaldehyde solution. The confocal slides were suspended with VECTASHIELD antifade mounting medium (H-1000) and fixed with cover slides. Cellular imaging with CNDs was excited with a diode-pumped solid-state (DPSS) laser at 405 nm, and the emissions from the CNDs were detected at 420–500 nm.

### 2.9. In Vitro PDT Test

The photodynamic therapy (PDT) efficacy of the CNDs was evaluated using A549 cells. Cell culture in the 96-well plate was carried out under the same conditions used for the cell viability test. After 1 d of cell culture time, the CNDs were added to each well at concentrations of 0.1, 0.5, and 1 mg·mL^−1^, and the cell culture plate was incubated at 37 °C in a humidified 5% CO_2_ incubator for 1 d. Afterwards, a 473 nm laser of 15 mW·cm^−2^ intensity was irradiated for 10 min. The cells were subsequently incubated at 37 °C for an additional 24 h. After replacing the cell medium solution with 100 µL of SFM, the CCK-8 assay solution was added to make 10% of the total concentration. After 2 h of incubation, the optical densities of each well were measured at 450 nm using a microplate reader.

## 3. Results and Discussion

**Figure 1:**
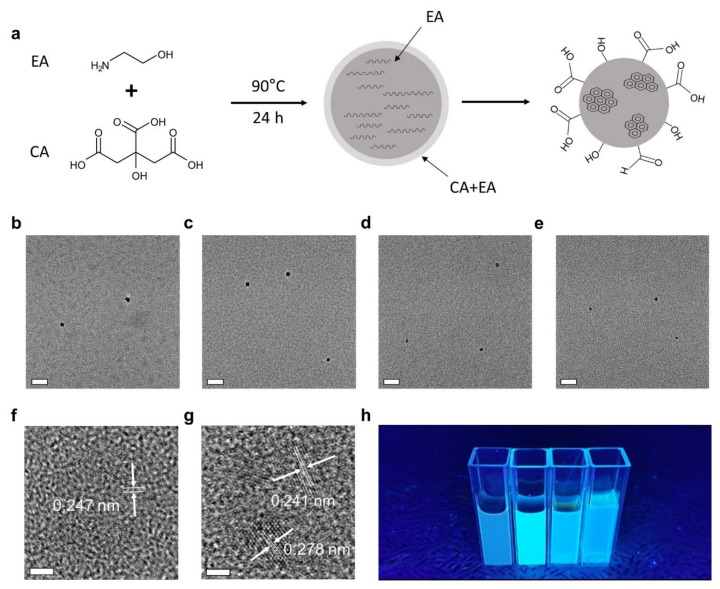
(**a**) Schematic illustration of the synthesis of CNDs. TEM images of (**b**) CND1, (**c**) CND2, (**d**) CND3, and (**e**) CND4. The scale bars represent 50 nm. (**f**,**g**) High-resolution TEM images of CDs. The scale bars represent 2 nm. (**h**) CND1–CND4 (left to right) under a 365 nm UV lamp.

The CNDs were synthesized via the oxidative carbonization of EA with a controlled amount of CA under ambient conditions. Generally, CA is used as the major precursor to form carbogenic cores and relatively a small amount of amine is needed to improve the colloidal stability and optical properties of CNDs [17,24,36,37]. Conversely, we have found that using EA as the major precursor (>70 mol%) and CA as an additive (<30 mol%) results in partially graphitic or polycrystalline CNDs at a low reaction temperature of 90 °C (Figure 1a). The as-synthesized CNDs with different molar percentages of EA (100, 95, 92, and 70 mol%) and CA (0, 5, 8, and 30 mol%) were labeled as CND1, CND2, CND3, and CND4, respectively.

TEM was performed to reveal the morphology and particle size of the CNDs. Figure 1b–e and Figure S1 shows TEM images of the CNDs with sizes ranging from 10–15 nm. As shown in Figure S2, regardless of the molar percentages of EA and CA, the size of the CNDs remains similar. The average size of the CNDs is approximately 9.91 ± 2.24, 10.25 ± 1.84, 10.37 ± 1.98, and 9.96 ± 1.52 nm in order. The size distribution and dispersity were further examined by HPLC and DLS. HPLC analysis shows that the retention time remains constant for all CNDs (Figure S3). DLS measurements show a nearly monodispersed size distribution around 15 nm (Figure S4), which confirms the high dispersity of the CNDs. Furthermore, the CNDs have highly negative zeta potentials (<−30 mV), which ensures the colloidal stability in water (Figure S5). The high-resolution TEM images show that the CNDs possess polycrystalline sp^2^ carbon clusters with the size ranging from 1–2 nm embedded in amorphous sp^3^ carbon matrices (Figure 1f,g) [38,39]. This amorphous nature of the CNDs was further confirmed by Raman spectroscopy (Figure S6).

**Figure 2:**
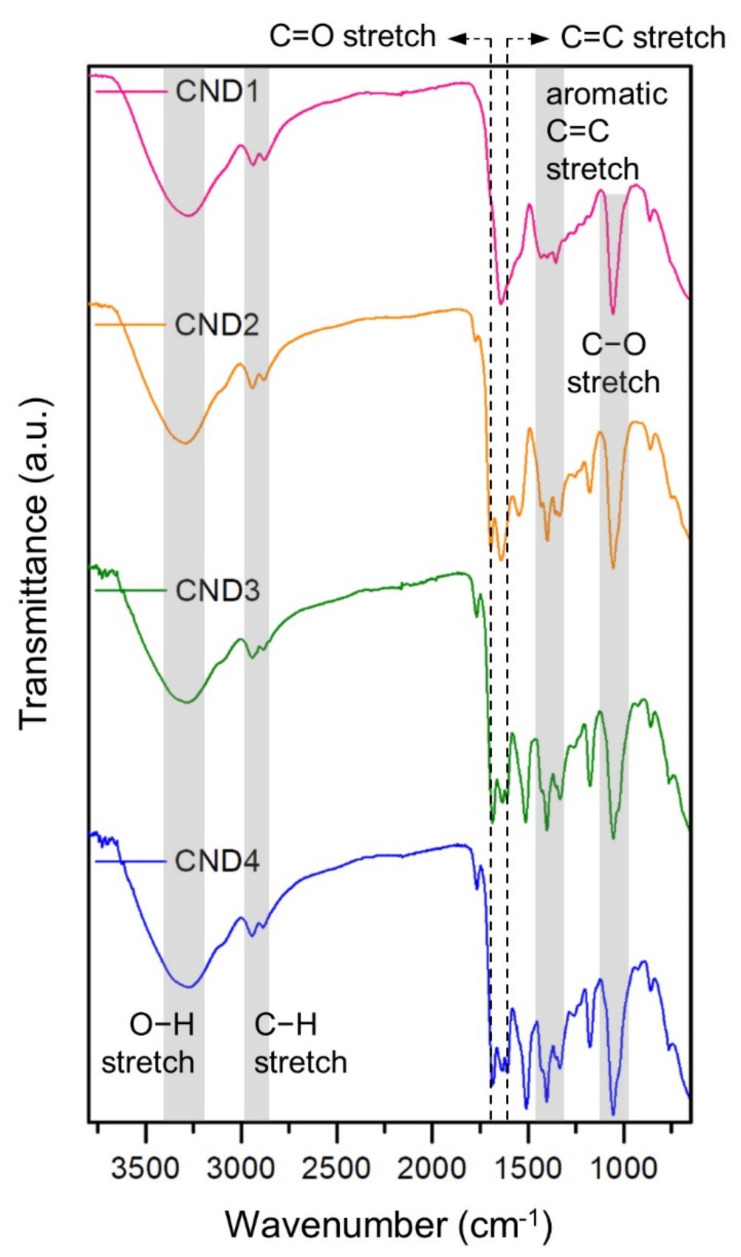
FT-IR spectra of the CNDs. The C=O (1693 cm^−1^) and C=C (1640 cm^−1^) stretch peaks are indicated by the dotted vertical lines.

FT-IR spectroscopy analyses were performed to investigate the surface chemistry of the CNDs. As shown in Figure 2, all the CNDs exhibited C-O stretching (1055 cm^−1^), aromatic C=C stretching (1350–1450 cm^−1^ and 1640 cm^−1^), C-H stretching (2850–2950 cm^−1^), and O-H stretching (3200–3400 cm^−1^) vibrational peaks, indicating the formation of polyaromatic structures and hydroxyl moieties in the CNDs. Additionally, a C=O stretching vibrational peak (1693 cm^−1^) was observed for CND2-CND4 with the intensity increasing toward the direction of increase in CA mol%. This indicates that a significant amount of carbonyl functional groups, mainly carboxylic acids, were formed on the surface of the CNDs after the addition of CA. With increase in the CA mol% (CND3 and CND4), a new C=C stretching vibrational peak (1609 cm^−1^) evolved while the original peak (1640 cm^−1^) remained preserved, indicating that the surface of the CNDs was modified to a large extent.

The C1s XPS data further showed that the C=O and C−O signals were significantly enhanced with increasing CA mol% (Figure S7). On the other hand, elemental analysis showed that the atomic (C, O, and N) compositions of the CNDs remained at a similar level regardless of the CA mol% (Table S1). Therefore, at low temperatures, the addition of CA results exclusively in the incorporation of carbonyl functional groups on the surface of the CNDs, rather than the formation of fully carbonized carbogenic structures with a high carbon content. The carbonyl functional groups are known to play a critical role in the occurrence of PL, and are hereafter referred to as CA-derived fluorescent moieties (CAFMs).

The formation mechanism of our CDs is not fully understood at this stage but may involve thermal oxidation of EA, likely to yield a variety of degradation compounds such as acetamides, oxamides, formates, oxalates, glycines, imidazoles, etc. [40]. These compounds are composed of carbonyls and dienes that are considered to be important for the formation of polyaromatic hydrocarbons. According to Chen et al., EA was critical for the formation of both amorphous (the mixture of sp^2^ and sp^3^) and graphite-like (sp^2^) carbons, which was confirmed by Raman spectroscopy [41]. This indicates that, in our case, the degradation compounds of EA may be decomposed and then reconstructed to form sp^2^ carbons of a conjugated π electron system, which is responsible for the (partially) graphitic structure and related optical properties of the CDs.

To investigate the optical properties of the CNDs, we conducted UV-visible absorption and PL spectroscopy measurements. As shown in Figure S11, the absorption spectrum of CND1 shows a broad absorption band (250 to 550 nm), arising from noncollinear π-plasmon transitions in the polyaromatic structures of the CNDs [42]. Another absorption band at ~400 nm could be assigned to the n-π* transition in the molecular energy state of CAFMs [15,16,17]. As soon as CA was added (CND2), a new absorption peak was observed at ~340 nm, indicating that CAFMs would provide certain intraband energy states. With a further increase in the CA mol% (CND3 and CND4), the absorption peak at 340 nm enhanced, which confirmed that these intraband energy states were strongly related to CA and CAFMs.

**Figure 3:**
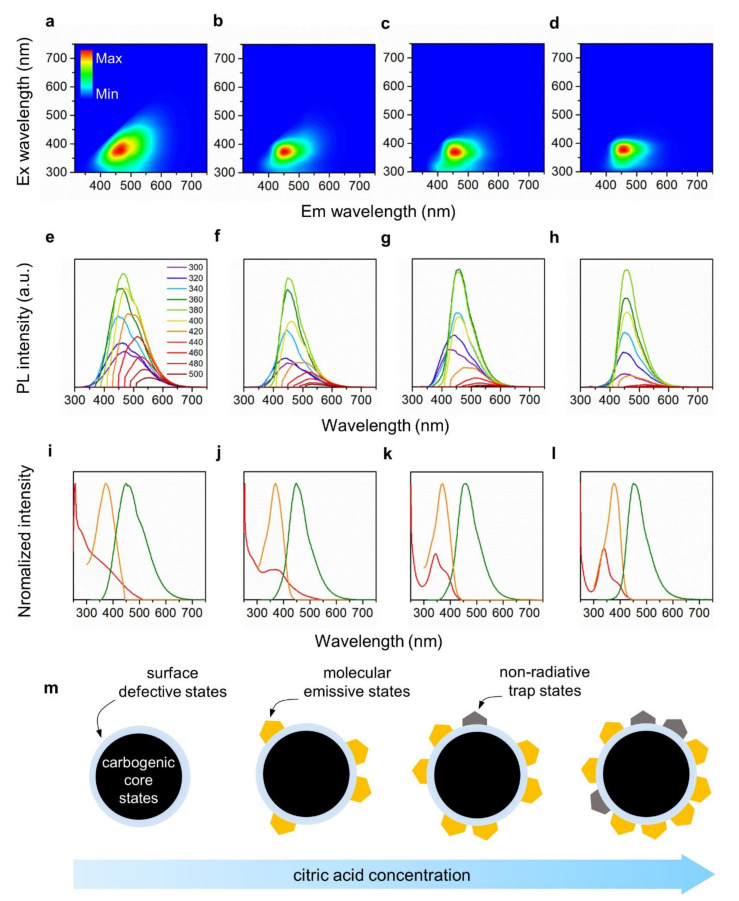
PL emission maps and spectra for excitation wavelengths of 300–600 nm. Emission maps: (**a**) CND1, (**b**) CND2, (**c**) CND3, and (**d**) CND4. Emission spectra: (**e**) CND1, (**f**) CND2, (**g**) CND3, and (**h**) CND4. Absorption (red), excitation (orange), and emission (green) spectra: (**i**) CND1, (**j**) CND2, (**k**) CND3, and (**l**) CND4. (**m**) Schematic illustration of the change in chemical structure with increase in the concentration of citric acid.

The PL emission contour maps in Figure 3a–d exhibit a broad emission peak centered at ~450 nm for all the CNDs. Although the peak emission wavelengths of the CNDs were almost the same, the detailed features of the emission spectra varied significantly with the CA mol%. As shown in Figure 3e, the emission spectra of CND1 change significantly with respect to the excitation wavelengths. This excitation dependency is frequently observed in common CNDs and is explained by the presence of various surface emissive (or defective) states [43,44,45]. In this case, the excitation dependency gradually vanished with the addition of CA. CND4 exhibited completely excitation-independent emissions with a relatively narrow bandwidth. These are generally considered as the characteristics of molecular fluorophores, which indicates that CAFMs were formed predominantly when higher CA mol% was used to create molecular emissive states (Figure 3f–h). The absolute quantum yields of the CNDs were ca. 5~15% under the 365 nm excitation (Table S2). Our CNDs also exhibited excellent photostability against ambiance and/or UV (365 nm) irradiation; the PL intensities were mostly maintained after 10 h (Figure S12).

Considering that the addition of CA mostly changed the surface structure of the CNDs, CAFMs are likely to be located on the surface of the CNDs. According to Song et al., the condensation of CA and ethylenediamine (EDA) resulted in blue-luminescent molecular fluorophores called IPCA [34]. Since EA is chemically similar to EDA, we suggest that CAFMs in CND2-CND4 play a similar role to IPCA. For instance, the strong carbonyl peaks of CAFMs previously observed in FT-IR and XPS were also found in IPCA due to carboxylic acid groups. Therefore, we conclude that CAFMs, being chemically similar to IPCAs, were formed on the surface of the CNDs, creating well-defined, molecular emissive states associated with the excitation-independent and narrow-bandwidth PL. As the molar percentage of CA increased, CAFMs and their molecular emissive states became dominant over the other PL origins. Consequently, the PL pathways in the CNDs converged to the molecular emissive states.

Interestingly, however, the PL intensity was not proportional to the CA mol% or the amount of CAFMs formed in the CNDs. As shown in Figure S13, CND2 exhibited the highest PL intensity, which decreased with increasing CA mol%. This indicates that the addition of CA could lead to the formation of not only CAFMs but also other chemical moieties with incomplete molecular structures that contain dangling bonds and radicals. These chemically incomplete moieties can create non-radiative trap states because of their instability and high electron affinity. To determine the presence of these non-radiative trap states, we compared the absorption, PL excitation, and PL emission spectra in Figure 3i–l. For CND3 and CND4, two distinct absorption peaks at 340 nm and 390 nm were observed, while the PL excitation peak was present only at 390 nm. This indicates that excitons generated by the 340-nm excitation decay through a non-radiative pathway, and the energy state associated with this excitation is a non-radiative trap state. Therefore, as the CA mol% increases, undefined molecular structures and the associated non-radiative trap states are formed along with CAFMs (Figure 3m). Since the FT-IR data implied that the C=C stretching peak (1609 cm^−1^) became more pronounced with increasing CA mol%, we deduced that these C=C bonds might play a central role in generating non-radiative trap states.

**Figure 4:**
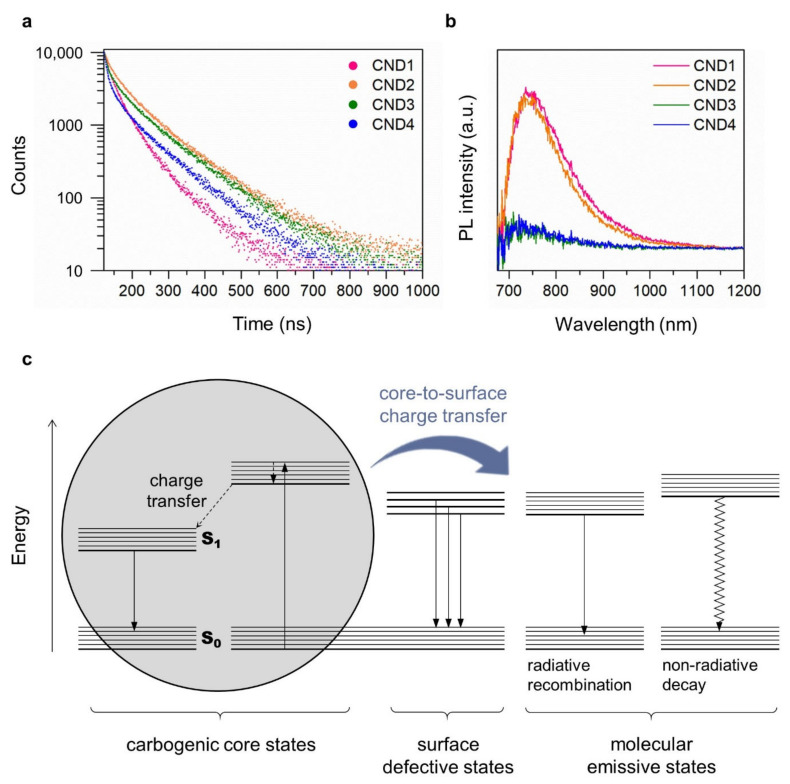
(**a**) TCSPC signals for CNDs recorded at detection wavelength of 450 nm with pump excitation wavelength of 374 nm. (**b**) NIR PL emission spectra of the CNDs. (**c**) Schematic illustration of emission pathways and related surface structures of the CNDs.

TCSPC measurements were conducted to further investigate the energy states of the CNDs. As shown in Figure 4a and Table 1, the TCSPC signals of the CNDs were fitted using a tri-exponential decay model. We found that the average fluorescence lifetime reached its highest value in CND2, and then decreased with increasing CA mol%, following the trend of the PL intensity. The average amplitudes of the long-lifetime components of CND2-4 were significantly higher than those of CND1, indicating that the densities of the long-lifetime excited states were increased by the addition of CA. Because the addition of CA is strongly related to the formation of CAFMs, we conclude that these long-lifetime excited states are directly associated with molecular emissive states. In contrast, the average amplitude of the short-lifetime components of CND4 was much higher than that of the other CNDs, indicating that non-radiative trap states were created in CND4 due to the presence of excess CA.

For CNDs in general, NIR PL has been considered to have different origins from the visible PL [46]. Although the exact mechanism remains unclear, it has been recently reported that polyaromatic core structures may play a major role in the occurrence of NIR PL [47]. In Figure 4b, CND1 and CND2 exhibit distinct NIR PL at around 750 nm, whereas CND3 and CND4 exhibit negligible NIR PL. This could be caused by an increase in the rate of charge transfer from the core states to the surface states. Since the density of molecular emissive states is higher in CND3 and CND4 than in CND1 and CND2, the photoexcited electrons would have a greater chance of being relaxed through molecular emissive states (visible emission) than through carbogenic core states (NIR emission). We present an energy diagram of the CNDs in Figure 4c. The carbogenic core states and the surface defective states, both created by the low-temperature carbonization of EA, were responsible for the occurrence of NIR PL and excitation-dependent visible PL, respectively. The molecular emissive states (associated with the excitation-independent and narrow-bandwidth PL) and the non-radiative trap states can be created by the addition of CA. The PL characteristics and associated surface chemistry of the CNDs (summarized in Table S3) are compared with other types of CNDs derived from CA and amine derivatives (Table 2).

**Figure 5:**
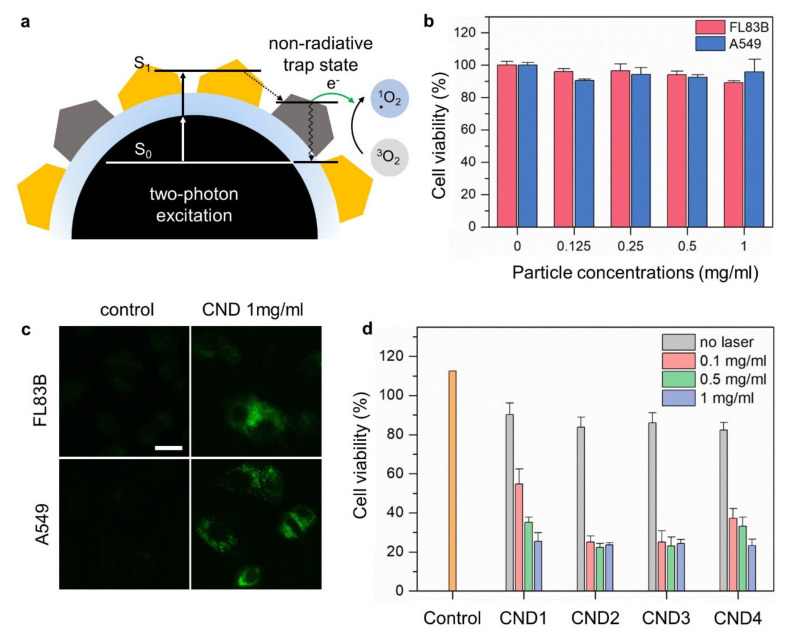
(**a**) Schematic illustration of the ROS generation by CNDs. (**b**) Cell viability test of the CNDs in FL83B and A549 cells using the WST-1 assay. (**c**) In vitro confocal microscopy images of FL83B and A549 cells with CNDs. The scale bar represents 25 μm. (**d**) Cell viabilities of A549 cells measured by the CCK-8 assay after photodynamic treatment at different concentrations of the CNDs. The error bars are obtained from three independent experiments and represent the standard deviation.

Finally, we investigated the behavior of photoexcited electrons in non-radiative trap states in CNDs (Figure 5a). As discussed earlier, these non-radiative trap states originate from incomplete chemical structures such as dangling bonds and radicals, which are capable of producing reactive oxygen species (ROS). To detect ROS generated by the CNDs, ROS assays were carried out using singlet oxygen sensor green (SOSG) as a ROS probe. In the presence of ROS, SOSG emits a green fluorescence similar to that of fluorescein, which exhibits PL at 525 nm. After exposure to UV irradiation (254 nm) in the presence of SOSG for 2 h, the PL intensities at 525 nm (in the aqueous solutions of the CNDs) were enhanced by ~2–3 times compared to pure water (Figure S14). Interestingly, CND2-CND4 produced more ROS than CND1, which further confirmed our hypothesis that non-radiative trap states created by the addition of CA were responsible for ROS generation.

Subsequently, we demonstrated apoptotic cell death induced by ROS generation to realize PDT based on our CNDs. Firstly, the cell viability of CNDs was investigated in normal musculus hepatocyte (FL83B) and lung epithelial carcinoma (A549) cells using the cell counting kit-8 (CCK-8) assay (Figure 5b). The cells were incubated for 1 d in a cell culture media containing CNDs with concentrations ranging from 0.125–1 mg·mL^−1^. The cell viabilities of the CNDs fell in the range of 90–100% at concentrations below 1 mg·mL^−1^ for both FL83B and A549 cells. Figure 5c shows confocal microscopy images of the FL83B and A549 cells after treatment with the CNDs to confirm their cellular internalization. The cells treated with the CNDs were green compared to the control group, which was not visualized. These results indicated that the CNDs were capable of penetrating into the cytosol through the cell membrane. Hence, CNDs can be used as a potential diagnostic and therapeutic agent.

The PDT efficacy of the CNDs at different concentrations was evaluated in vitro using A549 cells under 473 nm two-photon excitation. The experimental group treated with the CNDs showed a significant decrease in cell viability of ~25% (Figure 5d). CND2-CND4 with surface molecular fluorophores exhibited higher PDT efficacies than those exhibited by CND1. This is consistent with previous ROS data that indicated a central role of non-radiative trap states (induced by the addition of CA) in ROS generation.

## 4. Conclusions

In summary, we have successfully synthesized CNDs at relatively low temperatures (<100 °C) using EA as the major precursor and CA as an additive. The roles of EA and CA were qualitatively analyzed by controlling their molar percentages. The carbogenic core states and the surface defective states, created primarily through the carbonization of EA, were responsible for the occurrence of 750 nm NIR PL and excitation-dependent visible PL, respectively. In the presence of CA, CAFMs were formed on the surface of the CNDs, resulting in the formation of molecular emissive states. These states are associated with the occurrence of excitation-independent and narrow-bandwidth emissions. Furthermore, the addition of CA resulted in the formation of incomplete chemical moieties (comprising dangling bonds and radicals), which led to the creation of non-radiative trap states. These non-radiative trap states permit the generation of ROS. Using our CNDs, we demonstrated that PDT induced apoptotic cell death in A549 cells, which might be a useful treatment for cell carcinoma. We anticipate that these new insights into the energy states of CNDs will result in significant improvements in a variety of applications, ranging from photoelectrochemical and photocatalytic applications to biological labeling, biosensing, and medical treatment.

## Figures and Tables

**Table 1:** Fluorescence lifetimes of the CNDs.

Sample	*τ*_1_ (ns)	*A*_1_	*τ*_2_ (ns)	*A*_2_	*τ*_3_ (ns)	*A*_3_	*τ*_avg_ (ns)
CND1	4.05	58.96	10.45	29.12	1.18	11.92	3.65
CND2	5.03	30.50	12.66	63.44	1.39	6.07	6.47
CND3	4.19	24.54	12.49	69.27	0.84	6.18	5.32
CND4	3.91	24.96	11.71	60.67	0.88	14.37	3.58

**Table 2:** Brief literature review of CNDs derived from the mixture of CA and amine derivatives.

Precursors	Reaction Condition	Size (nm)	PL Ex/Em Wavelength	QY	Application	Ref.
Citric acid Ethanolamine	190 °C, 2 h	12.4 ± 5.6	376 nm/ 466 nm	11%	Subsurface tracer	[48]
Citric acid Ethanolamine	230 °C, 30 m	19	375 nm/ 455 nm	50%	−	[24]
Citric acid Ethylenediamine	140 °C, 5 h	2~6 (carbon core)	361 nm/ 442 nm	70% (relative)	−	[34]
Citric acid Ethylenediamine	120 °C, 15 h	3.5 ± 0.3	340 nm/ 440 nm	21.8% (relative)	−	[17]
Citric acid Triethylamine	160 °C, 4 h	1.7 ± 0.21	350 nm/ 437 nm	18.8%	Hg^2+^ detection	[49]
Citric acid Ethanolamine	90 °C, 24 h	10.25±1.84	360 nm/ 450 nm	15.24%	Bioimaging and PDT	This work

## Supplementary Materials

The following supporting information can be downloaded at: https://www.mdpi.com/article/10.3390/nano12010070/s1, Figure S1: TEM; Figure S2: Size distribution; Figure S3: HPLC; Figure S4: DLS; Figure S5: Zeta potentials; Figure S6: Raman spectroscopy; Figure S7: C1s XPS; Figure S8: N1s XPS; Figure S9: O1s XPS; Figure S10: XPS survey; Figure S11: UV-visible absorption spectroscopy; Figure S12: Photostability; Figure S13: PL intensity; Figure S14: SOSG PL; Table S1: Elemental analysis; Table S2: Absolute quantum yield; Table S3: PL/surface chemistry summary.
