# Supplementary material for: Radiative and Non-Radiative Decay Pathways in Carbon Nanodots toward Bioimaging and Photodynamic Therapy
Source: Nanomaterials (Basel). 2021 Dec 28;12(1):70. doi: 10.3390/nano12010070 (PMC8746803; doi:10.3390/nano12010070)
Supplement: Supplementary file 1 [file nanomaterials-12-00070-s001.zip › nanomaterials-1482201-SI.pdf]

Supplementary Materials

Radiative and Non-Radiative Decay Pathways in Carbon Nanodots toward Bioimaging and Photodynamic Therapy

Yujin Kim [1,†], Yoonsang Park [1,2,†], Seulgi Han [3,†], Wonchan Park [3], Mungu Kim [3], Kyunghwan Kim [4], Jinmyoung Joo [5], Sei Kwang Hahn [3,*] and Woosung Kwon [1,2,*]

1 Department of Chemical and Biological Engineering, Sookmyung Women's University, Seoul 04310, Korea; eugene5980@sookmyung.ac.kr (Y.K.); ypark@keti.re.kr (Y.P.)
2 Institute of Advanced Materials and Systems, Sookmyung Women's University, Seoul 04310, Korea
3 Department of Materials Science and Engineering, Pohang University of Science and Technology (POSTECH), Pohang 37673, Korea; seulgihan@postech.ac.kr (S.H.); mlblucky@postech.ac.kr (W.P.); kimmungu@postech.ac.kr (M.K.)
4 Department of Chemistry, Ulsan National Institute of Science and Technology (UNIST), Ulsan 44919, Korea; starcato@unist.ac.kr
5 Department of Biomedical Engineering, Ulsan National Institute of Science and Technology (UNIST), Ulsan 44919, Korea; jjoo@unist.ac.kr
* Correspondence: skhanb@postech.ac.kr (S.K.H.); wkwon@sookmyung.ac.kr (W.K.); Tel.:+82-54-279-2159 (S.K.H.); 82-2-2077-7398 (W.K.); Fax: 82-54-279-2399 (S.K.H.); 82-2-2077-7278 (W.K.)
† These authors contributed equally to this work.

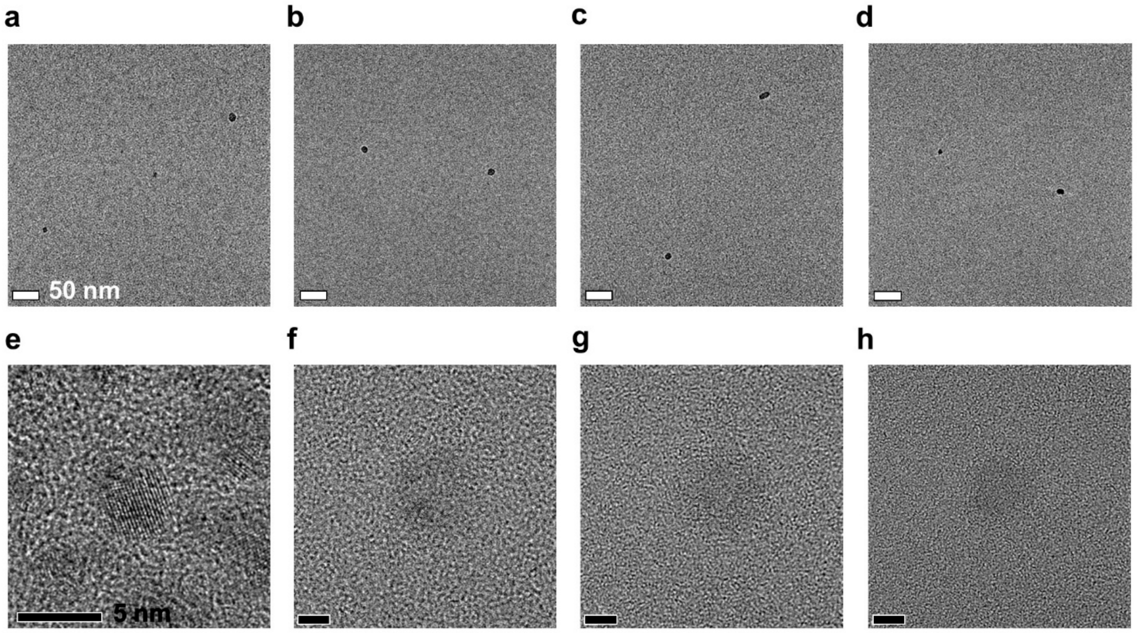

Figure S1. Additional (a-d) TEM and (e-h) HRTEM images of (a,e) CND1, (b,f) CND2, (c,g) CND3, and (d,h) CND4.

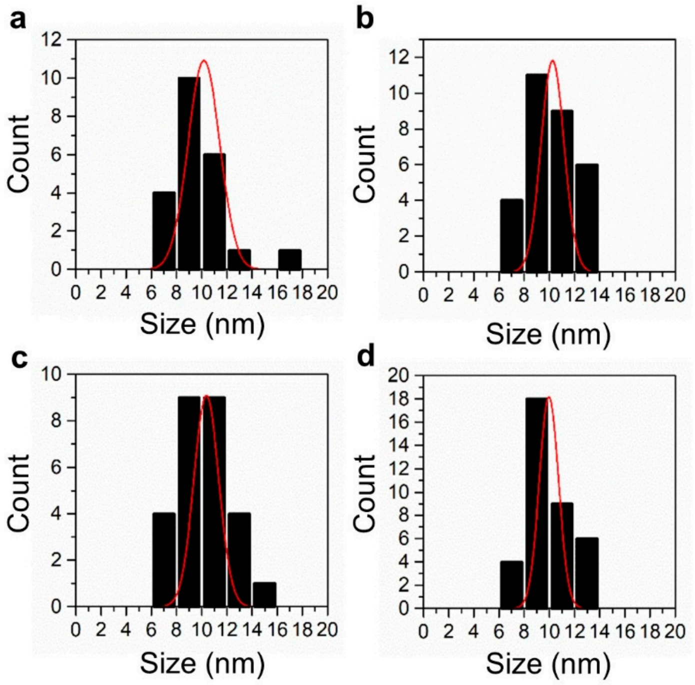

Figure S2. Size distributions of (a) CND1, (b) CND2, (c) CND3, and (d) CND4.

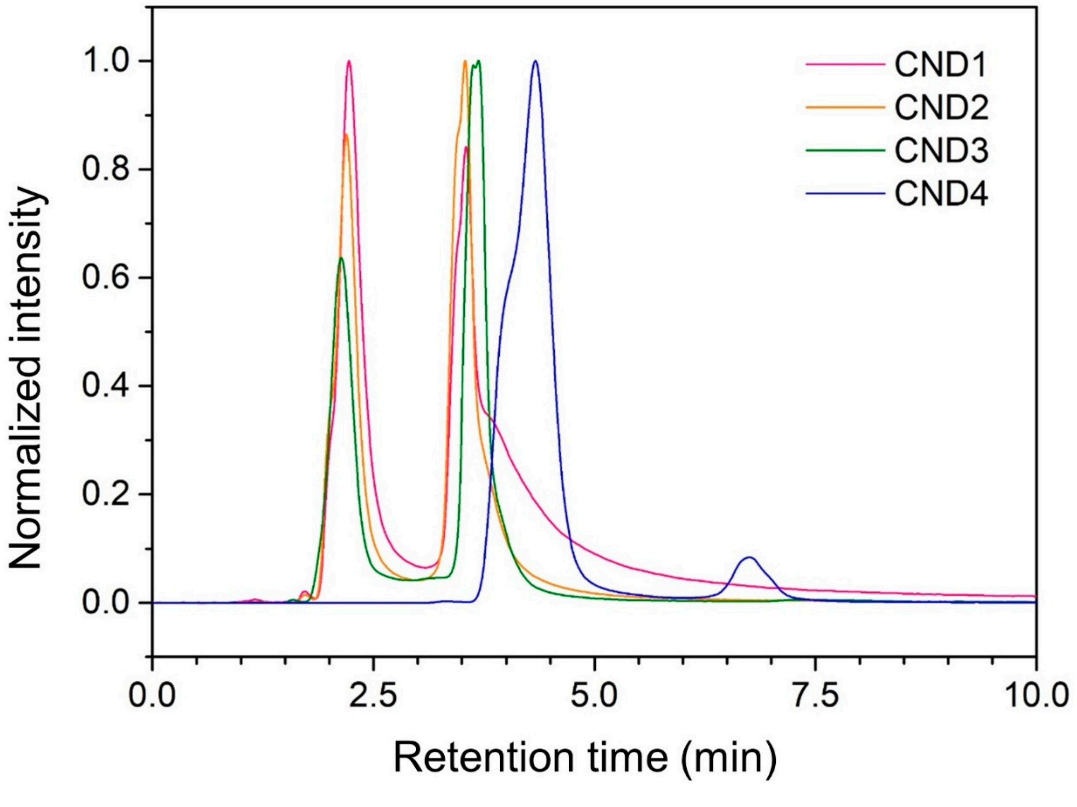

Figure S3. HPLC analysis of the CNDs monitored by the 350 nm UV absorption.

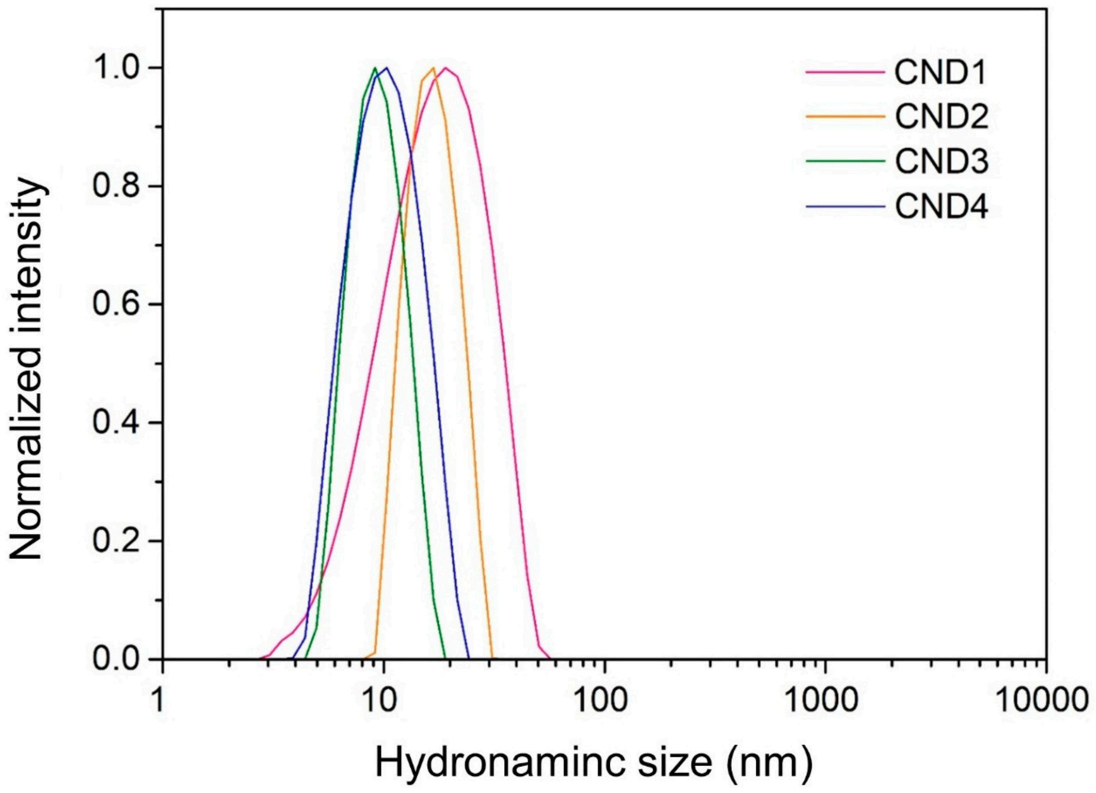

Figure S4. Size distributions of the CNDs measured by DLS.

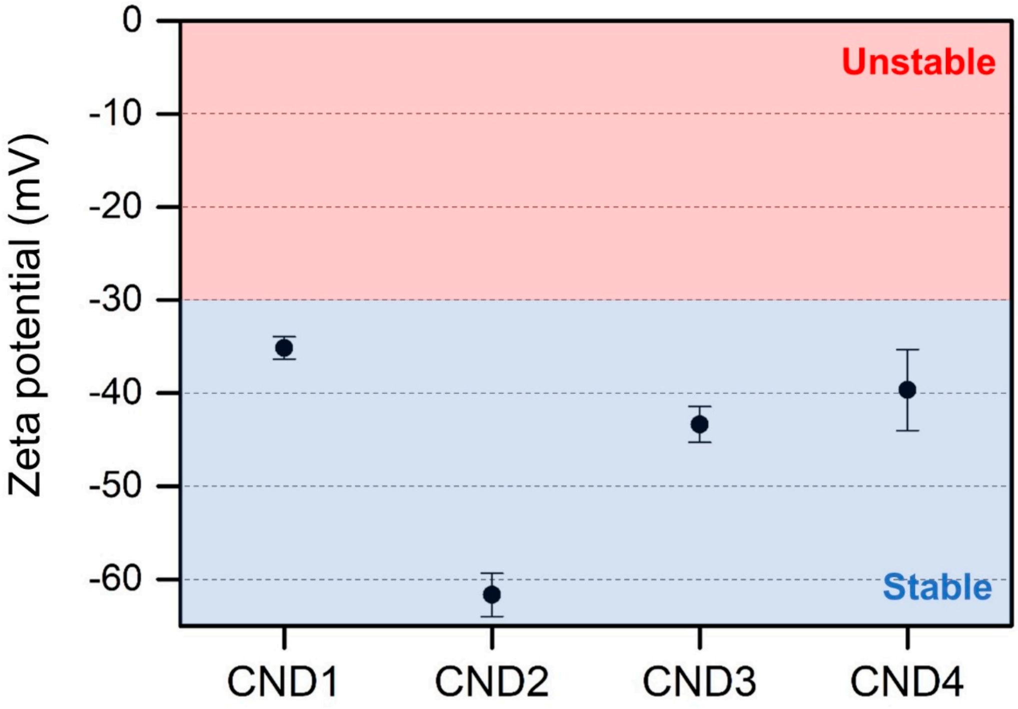

Figure S5. Zeta potentials of the CNDs are -35.16±1.20, -61.68±2.33, -43.36±1.92, and -39.66±4.35 mV in order. The error bars are obtained from five independent experiments and represent the standard deviation. As CA was added, surface carbonyl groups were significantly formed to impart a more negative zeta potential (CND2). However, excess CA would also lead to the formation of dangling/C=C bonds (as mentioned in the FT-IR and XPS analyses), compensating the decrease in zeta potential due to the surface carbonyl groups.

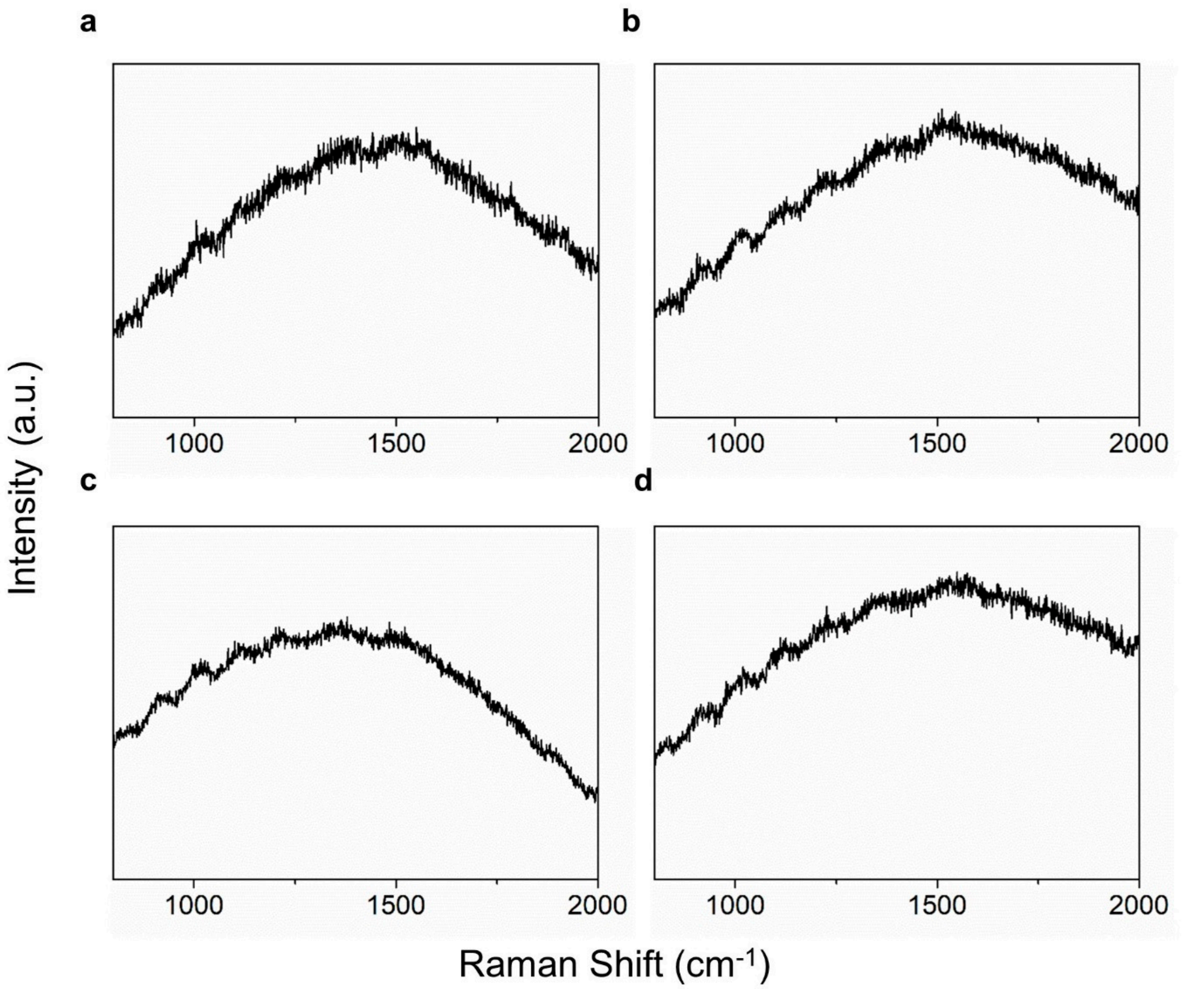

Figure S6. Raman spectra of (a) CND1, (b) CND2, (c) CND3, and (d) CND4.

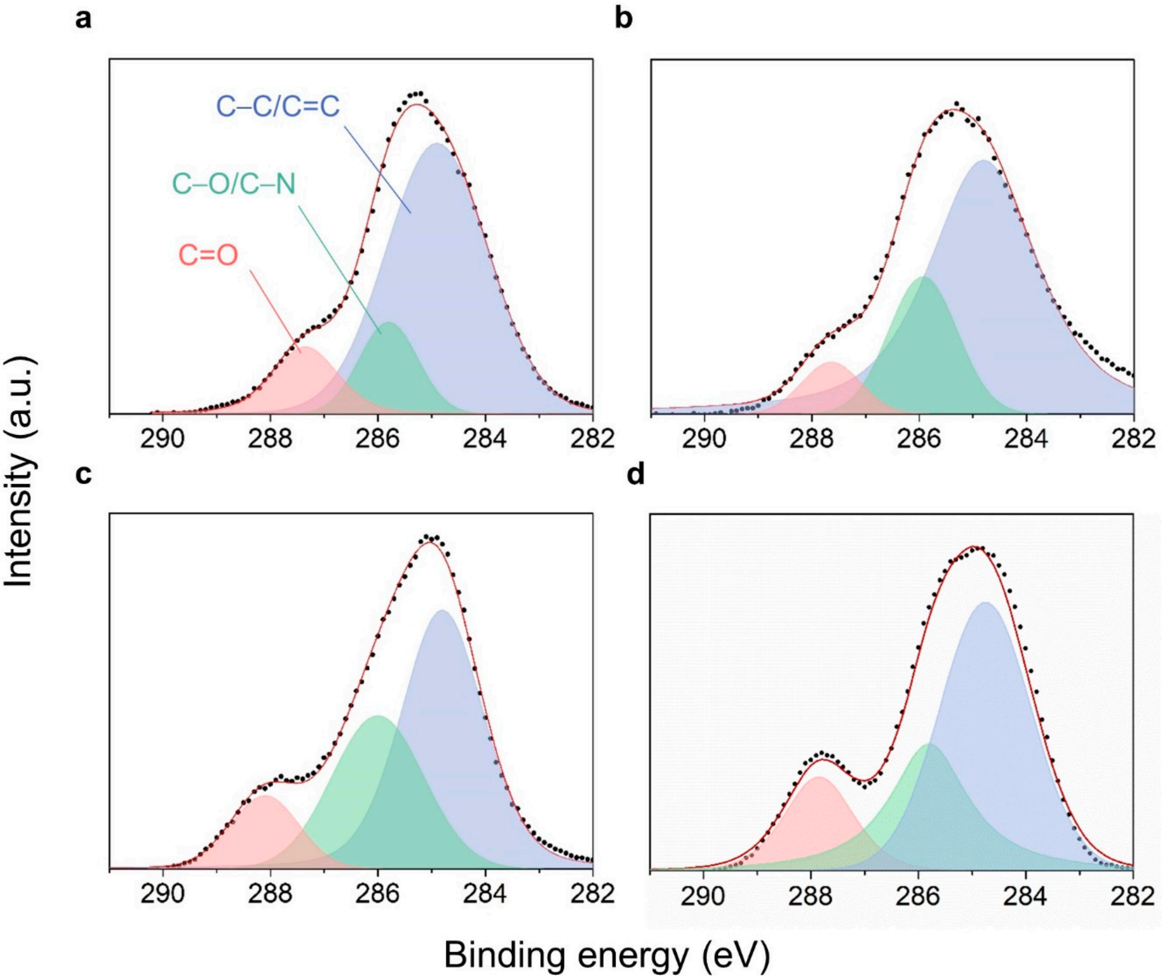

Figure S7. C1s XPS spectra of (a) CND1, (b) CND2, (c) CND3, and (d) CND4.

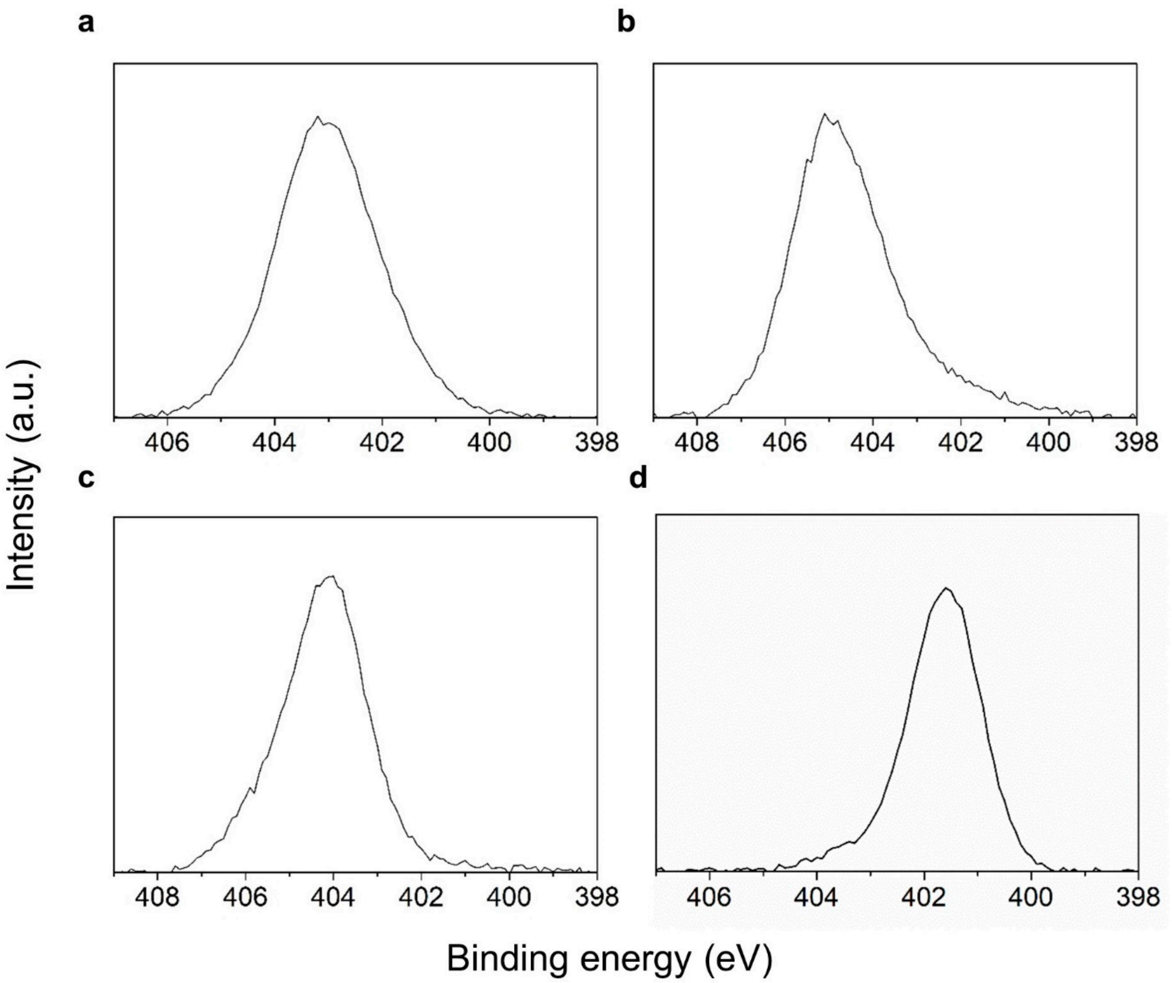

Figure S8. N1s XPS spectra of (a) CND1, (b) CND2, (c) CND3, and (d) CND4.

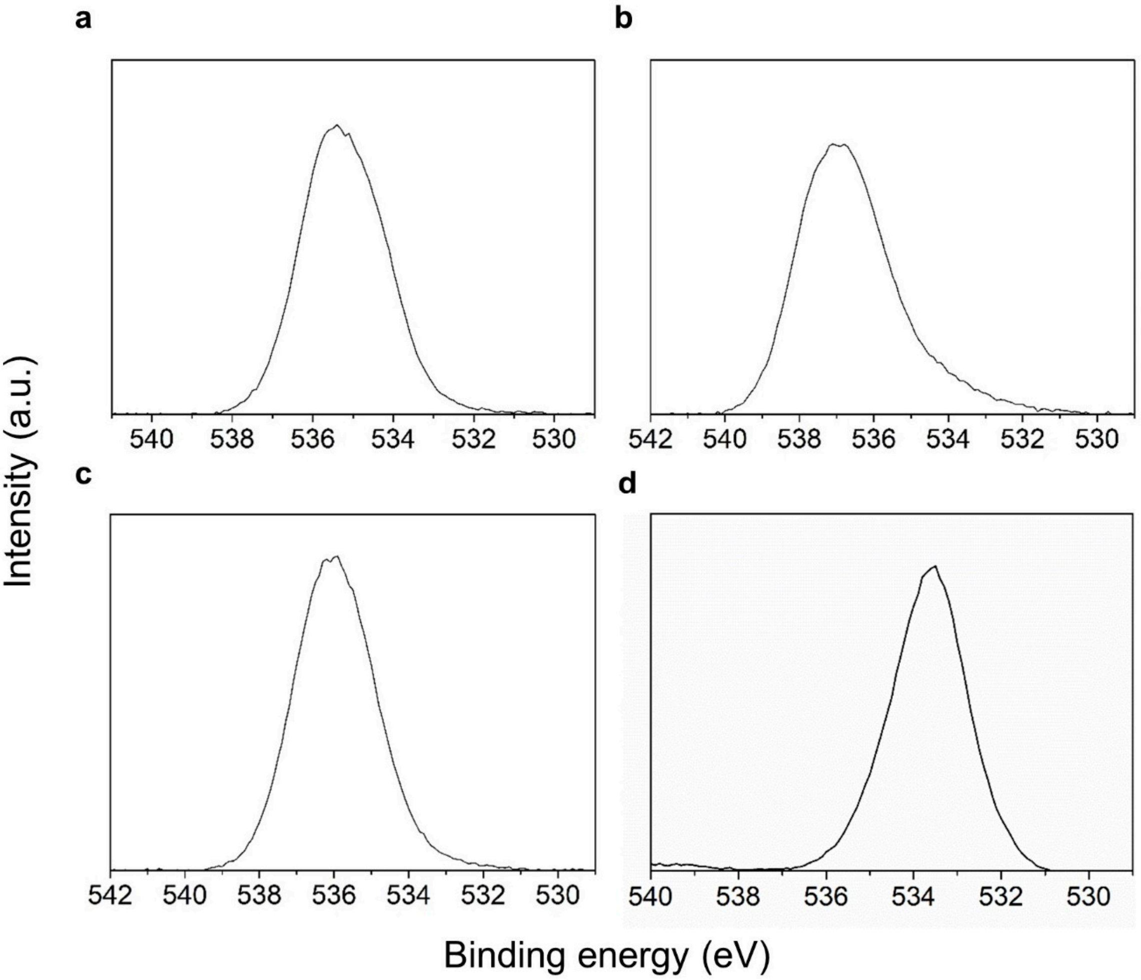

Figure S9. O1s XPS spectra of (a) CND1, (b) CND2, (c) CND3, and (d) CND4.

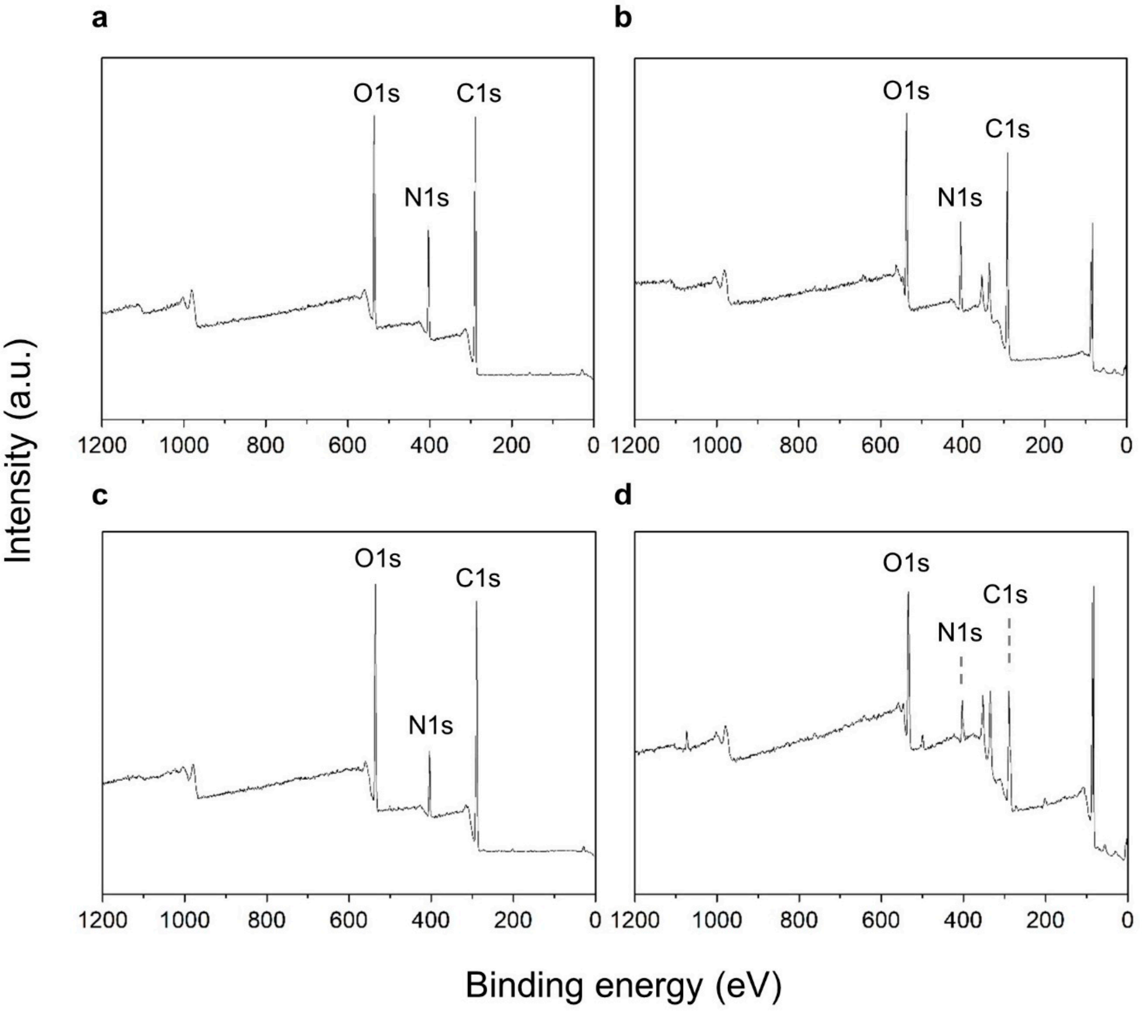

Figure S10. XPS survey spectra of (a) CND1, (b) CND2, (c) CND3, and (d) CND4.

Table S1. Elemental analysis data of the CNDs.

**Sample**	Atomic ratio (%)		
	C	O	N
CND1	64.64	20.24	15.12
CND2	63.48	22.43	14.08
CND3	68.91	21.65	9.44
CND4	64.25	25.73	10.01

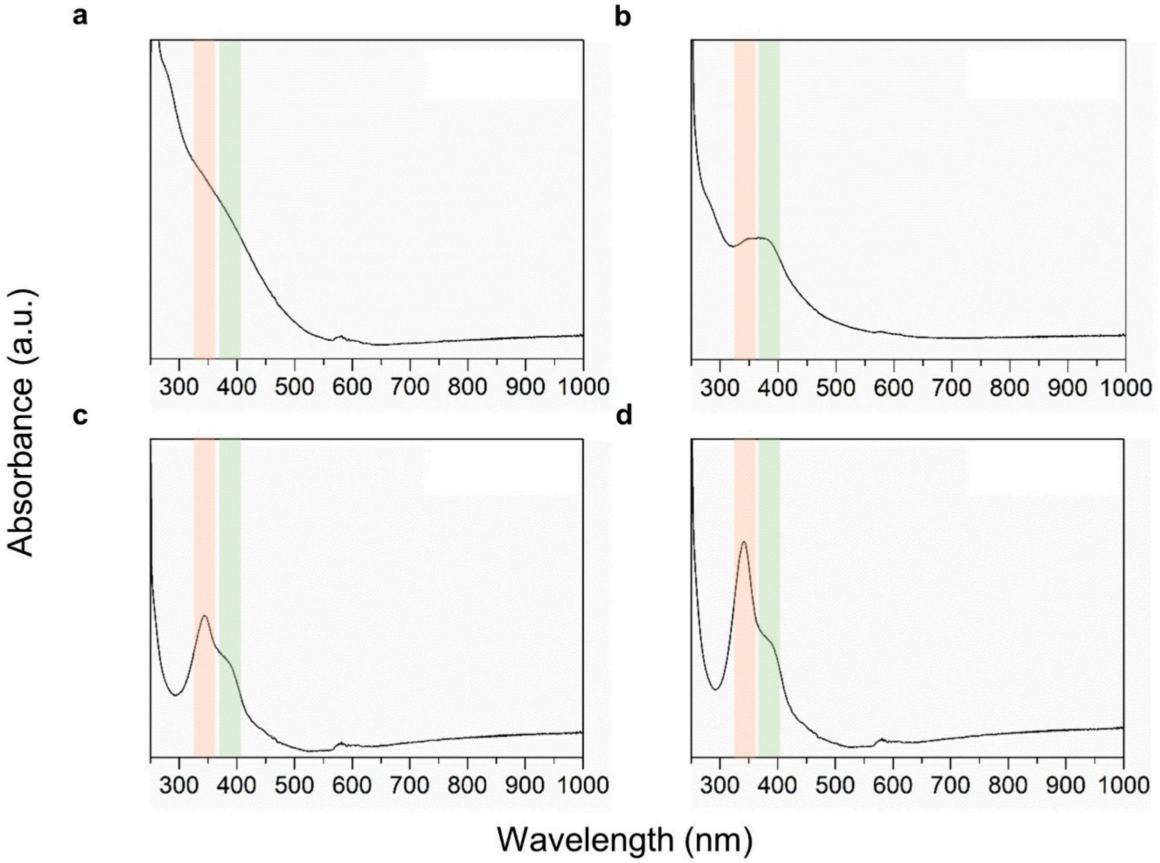

Figure S11. UV-visible absorption spectra of (a) CND1, (b) CND2, (c) CND3, and (d) CND4.

Table S2. Absolute quantum yields of the CNDs (excitation wavelength = 360 nm).

Sample	Absorptance (%)	Int. Quantum Efficiency (%)	Incident Light	Fluorescence	Scattering
CND1	8.079	6.647	4985.22	26.77	4582.48
CND2	10.721	15.239	4985.22	81.44	4450.77
CND3	20.092	10.694	4985.22	107.12	3983.59
CND4	15.906	4.824	4985.22	38.25	4192.27

Table S3. Summary of the PL characteristics and associated surface chemistry of the CNDs.

Sample	Molar percentages of CA (mol%)	Max. emission wavelength (nm)	Excitation dependency	avg. lifetime (ns)	PLQY (%)	Characteristic peaks	Recombination pathways
CND1	0	450	dependent	3.65	6.65	C–O (1055 cm^{-1})	surface state
CND2	5	450	dependent	6.47	15.24	C–O (1055 cm^{-1})<br>C=O (1693 cm^{-1})	surface state<br>+ molecular state
CND3	8	450	almost independent	5.32	10.69	C–O (1055 cm^{-1})<br>C=O (1693 cm^{-1})<br>C=C (1609 cm^{-1})	surface state<br>+ molecular state<br>+ trap state
CND4	30	450	independent	3.58	4.82	C–O (1055 cm^{-1})<br>C=O (1693 cm^{-1})<br>C=C (1609 cm^{-1})	surface state<br>+ molecular state<br>+ trap state

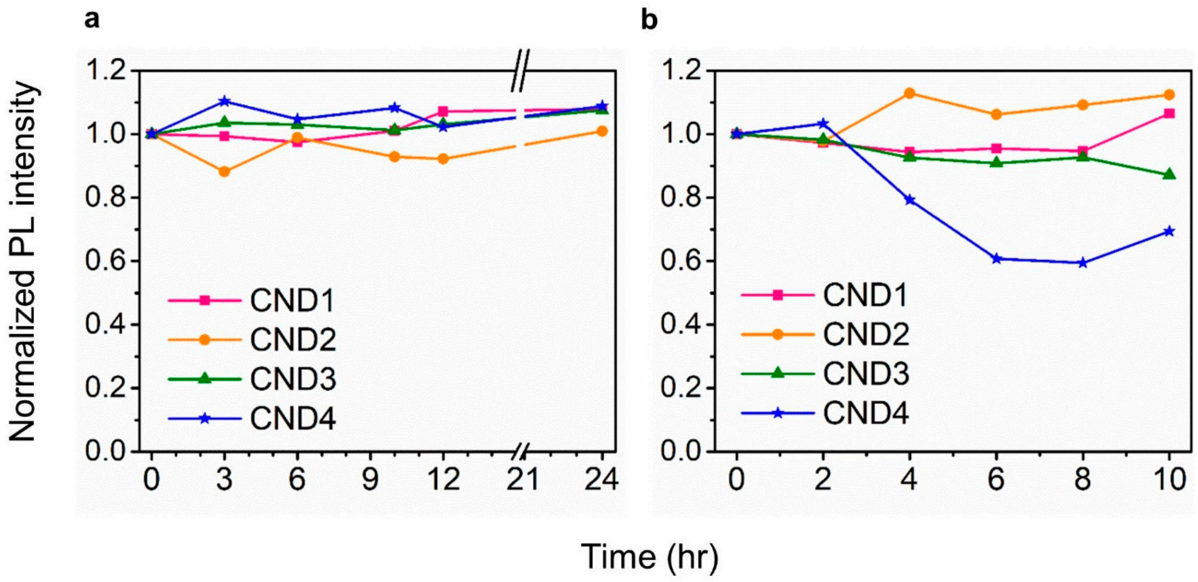

Figure S12. Photostability of the CNDs against (a) ambience and (b) UV irradiation. The PL intensities were measured at the excitation wavelength of 360 nm. All the CNDs except for CND4 exhibited excellent photostability against photobleaching. In the case of CND4, the PL intensity was decreased to 60%. This is because CND4 is majorly composed of molecular fluorophores that are more vulnerable to UV light than other fluorescent chemical structures.

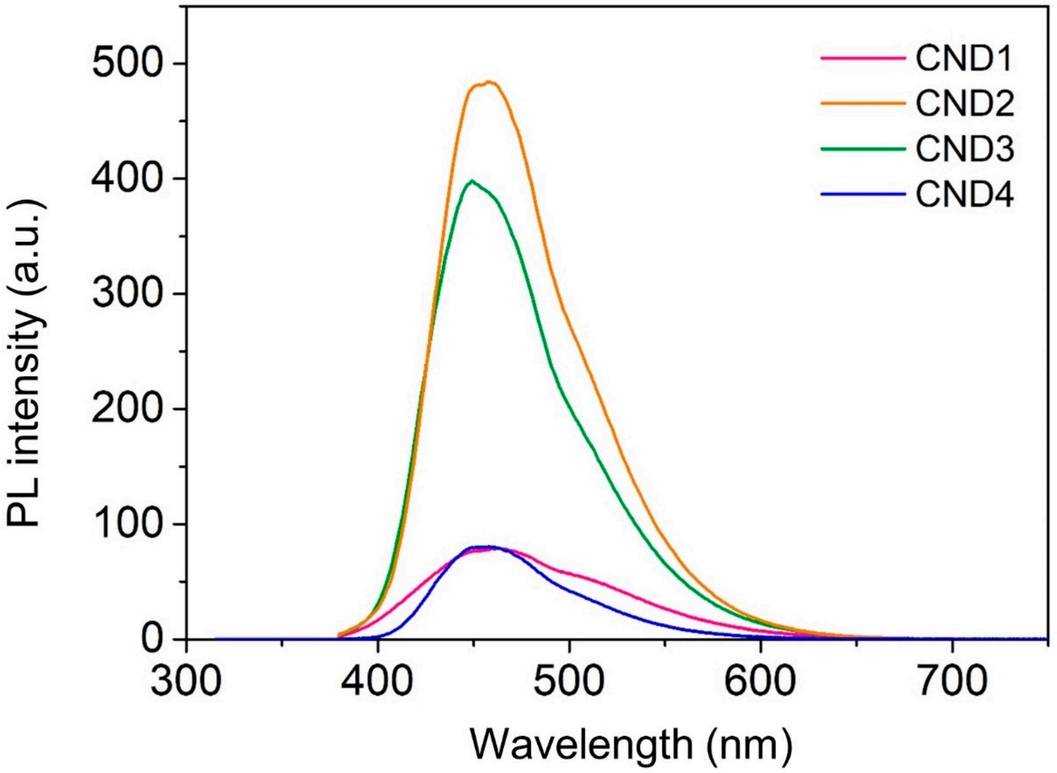

Figure S13. PL emission spectra of the CNDs (excitation wavelength = 360 nm).

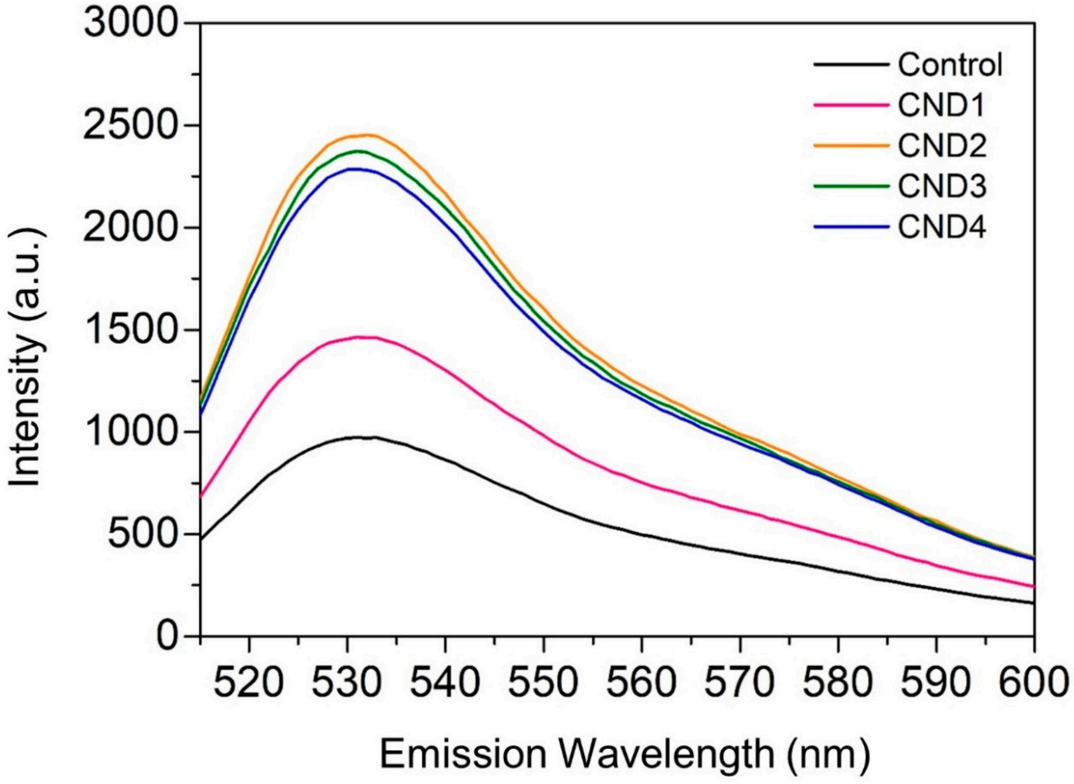

Figure S14. PL spectra of SOSG after 2 h of exposure to UV (254 nm, 8 W). The control experiment without the CNDs shows the base level of fluorescence due to singlet oxygen detection of SOSG.
